# Assessment of electrocardiogram interpretation competency among healthcare professionals and students of Ardabil University of Medical Sciences: a multidisciplinary study

**DOI:** 10.1186/s12909-022-03518-0

**Published:** 2022-06-09

**Authors:** Keyvan Amini, Alireza Mirzaei, Mirtohid Hosseini, Hamed Zandian, Islam Azizpour, Yagoob Haghi

**Affiliations:** 1 Department of Internal Medicine, School of Medicine. Fatemi Hospital, Ardabil University of Medical, Ardebil, Iran; 2 Students Research Committee, Department of Emergency Nursing, School of Nursing and Midwifery, Ardabil University of Medical Sciences, Ardabil, Iran; 3 Department of Critical Care Nursing, School of Nursing and Midwifery, Guilan University of Medical Sciences, Guilan, Iran; 4 Social Determinants of Health Research Center , Ardabil University of Medical Sciences, Ardabil, Iran; 5 Faculty of Medicine & Paramedical, Ardabil University of Medical Sciences, Ardabil, Iran

**Keywords:** Electrocardiography, Clinical skill, Healthcare professionals; students, Multidisciplinary care team

## Abstract

**Background:**

Electrocardiogram (ECG) interpretation is a core clinical skill that helps to rapid diagnosis of potentially life-threatening diseases. Misinterpretation of the electrocardiogram can lead to inappropriate clinical decisions with adverse outcomes. The main aim of this survey was to assess the competency of electrocardiogram interpretation and related factors among healthcare professionals and students of Ardabil University of Medical Sciences.

**Methods:**

This descriptive cross-sectional study included 323 staff and students of Ardabil University of Medical Sciences in northwestern Iran. Data were collected randomly from November to January 1400 using the Badell-Coll ECG Interpretation Competency Questionnaire and analyzed using SPSS V.14. Statistical analysis included descriptive statistics, independent t-test, ANOVA, Pearson correlation coefficient and multiple linear regression.

**Results:**

The results showed that the mean and standard deviation of electrocardiogram interpretation competency of health professional staff and students was 5.13 ± 2.25 (maximum score = 10). The large number of participants wasn’t able to identify normal sinus rhythm (*n* = 251, 77.3%), acute myocardial infarction (*n* = 206, 63.8%) and pathological Q waves (*n* = 201, 62.2%). The results of multiple linear regression showed that the variables of education level, self-assessment of electrocardiogram interpretation competence, work experience, and type of hospital were able to predict the competence of ECG interpretation in participants.

**Conclusions:**

Our findings showed that the participants’ level of electrocardiogram interpretation competency was low. Hence, regular, standard training and education are recommended. Also, managers and educators of the health system should consider the role of positive self-assessment and exposure to ECG interpretation in improving the competence of staff and students to interpret ECG.

## Introduction

A relatively small number of health professionals are confident in their independent electrocardiogram (ECG) interpretation. ECG interpretation is a cognitive skill that requires considerable time and effort to master [1]. Inappropriate interpretations, in turn, increase health care costs and can delay the admission process, imposing an unpleasant burden both on hospitals and patients [2].. To help solve the problem of declining literacy in ECG interpretation, the expected ECG interpretation competencies must be defined for all healthcare professionals [1]. Clinical competency is defined as an integration of skills, knowledge, attitude, values, and abilities that lead to effective or high performance in professional situations [3]. ECG interpretation competency involves familiarity with ECG interpretation, being skilled in lead placement, continuous monitoring, and having a proper attitude toward ECG interpretation [4]. People competent in ECG interpretation are those who (1) Have sufficient knowledge about the pathophysiological mechanisms of common and rare ECG abnormalities; (2) Are highly skilled in normal and abnormal patterns; (3) Ability to accurately judge whether the ECG recording quality is acceptable for interpretation; and (4) Have a strong understanding of scientific evidence that forms the basis of various ECG diagnoses [1]. The competency of health professionals, such as physicians, nursing teams, and prehospital emergency care staff, to record and interpret ECGs for the diagnosis of pathological disorders can help prevent heart disorders and reduce mortalities [5]. Shortcomings in ECG interpretation proficiency are not only due to the failure of individual learners. During the relatively short time allotted for formal medical education, learners are expected to study a wide range of subjects and acquire a variety of clinical skills [1]. Healthcare providers (for example, physicians, nurses, anesthetics, and paramedics who perform ECG) who work in environments where ECG interpretation may change patient management must be qualified to interpret ECG [1, 5].

An electrocardiogram (ECG) is a non-invasive test that monitors heart rhythm and electrical activity. It is considered the first diagnostic instrument for chest pain, as it allows specialists to assess any risks and symptoms [2]. According to the official statistics published by the Iranian Ministry of Health and Medical Education (MOHME), 33–39.3% of deaths in the country are due to coronary heart disease. This figure is forecasted to rise to 44.8% by 2020 [5]. ECG recording is necessary for all hospital units, as it helps diagnose conduction and electrical disorders of the heart and forecast the risk of such diseases [6]. This test has remained a cornerstone of medical practice for nearly one century [1]. International guidelines for Cardiopulmonary Resuscitation (CPR) and Emergency Cardiovascular Care (ECC) show that the correct and rapid interpretation of cardiac arrhythmias leads to the early identification of proper treatment and increases patient survival [7–9]. Although ECG interpretation is an essential skill for health professionals, there is still a gap in standard training programs and evaluation strategies to achieve and evaluate competency in ECG interpretation [10].

An ECG is sometimes the only and most efficient way of diagnosing life-threatening conditions, thereby enabling the provision of timely emergency care. The functional strength of a 12-lead ECG, however, depends on the physician’s ability to interpret the test correctly [11]. The diagnosis of arrhythmias by a physician, who is usually the first clinical contact in primary healthcare, can be beneficial to the patient [12]. Moreover, correct ECG interpretation during an emergency situation can help the physician eliminate possible non-cardiac causes of chest pain, such as gastrointestinal, esophageal, and musculoskeletal disorders or panic attacks, thus allowing proper patient management [13]. Research shows that the knowledge of medical and nursing staff in ECG interpretation is often limited due to the lack of inadequacy of training [14, 15].

Given that nurses are the health team specialists who are constantly present at the patient’s bedside, their ability to detect normal and pathological ECGs is of fundamental importance [6]. The accurate interpretation of ECG is a crucial skill for nurses and EMS staff, who care for patients in ECG emergencies [5, 16]. In particular, nurses should be able to record and interpret a 12-lead ECG correctly so that treatment can begin as soon as possible and better clinical outcomes can be achieved. Therefore, nursing staff must be able to identify the specific abnormalities of ECG signs and relate them to clinical conditions [14]. Rahimpoor et al. conducted a study to compare the ability to interpret ECGs among emergency nurses and EMS staff. They found that ECG nurses are more qualified in interpreting ECGs than prehospital emergency staff [5].

Competency in ECG interpretation is not a universal skill, and the barriers to achieving ECG fluency are rooted at multiple levels of medical curricula. Stopa et al. evaluated ECG interpretation competency among medical, nursing, and paramedical students in Poland and found that the overall score of the medical students was higher than that of the nursing and paramedical students [17]. Alghamdi et al. also conducted a study to evaluate ECG interpretation, address the nature of errors, and analyze the factors that determine the accuracy of ECG interpretation among medical students in year six of their program and interns at Taif University in Saudi Arabia. They found that the overall performance of medical students and interns was average [18]. In addition to these findings, other evidence suggests that the ECG interpretation competency of students is unsatisfactory [17, 19]. ECG interpretation can be improved by organizing ECG training courses [1]. ECG interpretation competency is a major challenge for medical educators. Historically, educators have relied on traditional methods of textbook reading or face-to-face interaction between teacher and student [1]. Recent research has examined and compared different teaching techniques, including self-directed learning (such as online courses or modules), lecture-based or workshop-based formats, and peer training in small groups [1, 4, 7, 11, 12]. ECG interpretation skills need to be continuously strengthened to preserve content. Thus, medical educators are not only challenged to provide effective training methods but also have the task of finding a training approach that helps ensure enduring sustainable progress [1, 5].

According to the official statistics of the Iran Ministry of Health, 33 to 39.3% of deaths are due to coronary artery disease and are anticipated to reach 44.8% by 2020 [5]. Therefore, it is necessary for health professionals and students involved with patients to be competent to receive and interpret ECGs. Healthcare providers and students can forecast any emergency care and provide the necessary interventions if they can independently interpret a 12-lead ECG. There are a limited number of studies that have assessed the competence of ECG interpretation for health professionals and students. In fact, in Iran, only one study has evaluated and compared the ECG interpretation of hospital emergency and medical emergency (EMS) nurses [5]. Therefore, this study aims to assess the competency of healthcare professionals and students in interpreting ECGs.

## Methods

### Study design

This cross-sectional, descriptive, correlational study was conducted to evaluate the competency of medical staff and students of Ardabil University of Medical Sciences in ECG interpretation.

### Study setting and participants

The study population of this study included all the interns and staff of medical centers and prehospital emergency staff in the city of Ardabil in northwestern Iran. The city of Ardabil consists of 12 EMS centers and 5 hospitals (1 hospital for heart specialization and 4 general and referrals hospitals). Inclusion criteria involved: qualified with a bachelor’s or master’s degree in nursing, associate’s or bachelor’s in a medical emergency; associate’s or bachelor’s degree in anesthesiology, PhD degree in medical and all the medical and paramedical students were in who doing their internship. Participants who did not wish to continue with the study and those whose questionnaires were incomplete were excluded from the study. In order to reduce the coercion and bias of faculty members and students, a space was created that did not threaten the relationship of trust between them. At all stages of the study, students were morally and methodologically protected by the research team. Thus, high standards of ethical actions were obtained in the study. Based on the initial estimates, the total population size was 1995. Using Cochran’s formula with an error level of 5%, the sample size was estimated at 323 [20]. In the first stage, the population was divided into homogeneous groups based on specific criteria. In the second stage, eight categories were determined based on the type of samples; the categories included medical interns, clinicians, senior nursing students, nursing staff working in teaching hospitals, EMS students, EMS staff, anesthesiology students, and anesthesiologists. The sample size for each group was calculated considering the proportion of that population from the total population using the following formula [21]:

$$n=\frac{N\kern0em {z}^2{s}^2}{N\kern0em {d}^2+{z}^2{s}^2}$$

The sample sizes included 37 medical students for the first category, 45 medical staff for the second category, ten nursing students for the third category, 194 nursing staff for the fourth category, eight EMS students for the fifth category, 12 EMS staff for the sixth category, eight anesthesiology students for the seventh category, and nine anesthesiologists for the eighth category. Hospitals and prehospital emergency services in Ardabil were selected, and random sampling was used to select participants from the interns, medical center staff, and prehospital emergency staff in proportion to their population sizes.

### Data collection

The data collection instrument in this study was a questionnaire consisting of two parts. The first part included the students’ and health professionals’ demographic and professional information, including educational level, age, gender, job title, work experience and history of internship in medical centers and hospital emergency services, participation in ECG interpretation courses, last ECG interpretation course date, ECG training method used in the courses, and duration of the ECG interpretation course. The second part was a specific multiple-choice questionnaire on ECG interpretation competency, including 12 items developed by Coll-Badell et al. in Spain to measure health staff’s ECG interpretation competency. The participants were asked to select the best answer, and if they did not know the answer to any of the items, to select the relevant option. This questionnaire has been translated into Persian and utilized in Iran by Rahimpoor et al. [5]. According to the developers of the questionnaire, the tool has a normal scoring method. One point is given to each correct answer, and the wrong answers or ‘I don’t know’ responses do not receive any points. Participants’ scores were calculated by counting the correct answers and converting the total score to 10 (to facilitate quantification and comprehension). The eligibility score was set from 0 to 10. An average score of 7.5 or above showed good competency in ECG interpretation. Cronbach’s alpha coefficient for the ECG interpretation competency questionnaire in the study of Coll-Badell [22] was 0.86, and in the study of Rahimpoor et al., [5] was 0.71. Its Cronbach’s alpha coefficient was 0.65 in the present study.

### Statistical analyses

The collected data were analyzed using IBM SPSS Statistics v 14 (SPSS Institute; The IBM Corporation, Somers, NY). Statistical tests included descriptive statistics, independent t-test, one-way ANOVA, Pearson correlation coefficient and multiple linear regression. *P* values less than 0.05 were considered statistically significant.

## Results

### Demographic characteristics of the participants

The results of our study (Table 1) indicated that 45.8% of the participants (*n* = 148) were in the age group of 20–28 years. 62.5% (*n* = 202) of the samples were female, and 67.5% (*n* = 218) were undergraduates. 60.1% of the participants (*n* = 194) were nursing. 34.4% of the participants (*n* = 111) have already passed the electrocardiogram interpretation retraining course, and the majority of them (66.9%, *n* = 216) have participated in these courses in person. The mean and Standard Deviation for work experience, the last time of the retraining period, and the number of ECGs recorded in each shift were 5.94 (SD = 5.18), 2.41 (SD = 1.89) and 1.81 (SD = 2.19), respectively. About 48.0% of the participants (*n* = 155) considered interpreting electrocardiogram as Job Duty. The majority of participants (53.9%, *n* = 174) rated their eligibility for electrocardiogram interpretation as moderate. About 20.4% (66 people) of the participants had experience working in the CCU, and 25.7% of participants (83 people) were working internships in specialized hospitals.

**Table 1:** Demographic characteristics of the participants (*N* = 323)

Variables		N (%)	Mean (SD)
Age group	20–28 years	148 (45.8)	
	29–37 years	137 (42.4)	
	38–45 years	30 (9.3)	
	46–53 years	8 (2.5)	
Gender	Male	121 (37.5)	
	Female	202 (62.5)	
Educational Level	Associate Degree (AD)	11 (3.4)	
	Bachelor of Science (BSc (	218 (67.5)	
	Master of Science (MSc)	14 (4.3)	
	PhD	80 (24.8)	
Job Title	Medical student	37 (11.5)	
	Physicians	45 (13.9)	
	Nursing student	10 (3.1)	
	Nurse	194 (60.1)	
	EMT student	8 (2.5)	
	EMT	12 (3.7)	
	Anesthesia student	8 (2.5)	
	Anesthesia staff	9 (2.8)	
Has taken an ECG course?	*Yes*	111 (34.4)	
	*No*	212 (65.6)	
Type of training	Online	79 (24.5)	
	Face-to-face	216 (66.9)	
	Partial face-to-face	28 (8.7)	
Experience			5.94 (5.18)
Last training time Years since taking the course			2.41 (1.89)
How many times do you record an ECG during a work shift?			1.81 (2.19)
Is ECG interpretation one of your tasks?	*Yes*	155 (48.0)	
	*no*	168 (52.0)	
Self-assessment of the ECG interpretation competency	Weak	69 (21.4)	
	Moderate	174 (53.9)	
	Good	67 (20.7)	
	Very Good	13 (4.0)	
Do you have experience working in the coronary care unit (CCU)?	*Yes*	66 (20.4)	
	*No*	257 (79.6)	
Type of hospital	Hospital (heart specialization)	83 (25.7)	
	General Referral hospital	180 (55.7)	
	Referral hospital (nonheart specialization)	60 (18.6)	

### Participants’ ability to interpret electrocardiograms

Participants’ ability to interpret the ECG is presented in Table 2. The mean score obtained by the participants was 5.25 2. 2.25. More than 60% of participants were able to diagnose atrial flutter (*N* = 225, 69.7%), supraventricular tachycardia (*N* = 214, 66.3%), grade 3 heart block (*n* = 214, 66.3%) and ventricular tachycardia (*n* = 203, were 62.8%). A significant proportion of participants were unable to detect normal sinus rhythm (*n* = 251, 77.3%), acute myocardial infarction (*N* = 206, 63.8%) and pathological Q waves (*n* = 201, 62.2%).

**Table 2:** Capability of the participants for electrocardiogram interpretation (*N* = 323)

	N (%)	Mean (SD)
**Rhythm 1 – Atrial flutter**		
Incorrect	98 (30.3)	
Correct	225 (69.7)	
**Rhythm 2 – Ventricular fibrillation**		
Incorrect	**109 (33.7)**	
Correct	214 (66.3)	
**Rhythm 3 – Atrial fibrillation**		
Incorrect	151 (46.7)	
Correct	172 (53.3)	
**Rhythm 4 – Pathological Q wave**		
Incorrect	201 (62.2)	
Correct	122 (37.8)	
**Rhythm 5 – Atrioventricular third-degree** bundle branch block		
Incorrect	109 (33.7)	
Correct	214 (66.3)	
**Rhythm 6 – Ventricular tachycardia**		
Incorrect	120 (37.2)	
Correct	203 (62.8)	
**Rhythm 7 – Acute myocardial infarction**		
Incorrect	206 (63.8)	
Correct	117 (36.2)	
**Rhythm 8 – Normal ECG**		
Incorrect	251 (77.7)	
Correct	72 (22.3)	
**Rhythm 9 – Ventricular extrasystole**		
Incorrect	159 (49.2)	
Correct	164 (50.8)	
**Rhythm 10 – Atrial tachycardia**		
Incorrect	167 (51.7)	
Correct	156 (48.3)	
**Score**		5.13 (2.25)

### Associations between the participants’ demographic characteristics and capability for electrocardiogram interpretation

The results showed that level of education, job title, take an ECG course, accepting electrocardiogram interpretation as a task, self-assessment of electrocardiography interpretation competency, having work experience in CCU, and type of hospital had a statistically significant relationship with the total score of ECG interpretation competency of healthcare professionals and students. (*P* < 0.05) **(**Table 3**).**

**Table 3:** Associations between the participants’ demographic characteristics and capability for electrocardiogram interpretation (*N* = 323)

Variables		Mean (SD)	*P* value
Age	20–28 years	5.06 (2.30)	F = 0.156 *p* = 0.92
	29–37 years	5.21 (2.26)	
	38–45 years	5.03 (2.17)	
	46–53 years	5.37 (1.92)	
Gender	Male	5.28 (2.37)	t = 0.891 *p* = 0.373
	Female	5.04 (2.18)	
Educational Level	Associate degree (AD)	3.00 (2.56)	F = 11.10 *p* < 0.001
	Bachelor of Science (BSc (	4.84 (2.15)	
	Master of Science (MSc)	5.35 (1.69)	
	PhD	6.17 (2.18)	
Job Title	Medical student	5.86 (1.97)	F = 6.400 *p* < 0.001
	Physicians	6.37 (2.30)	
	Nursing student	3.60 (1.57)	
	Nurse	5.04 (2.06)	
	EMT student	2.50 (2.97)	
	EMT	4.66 (2.30)	
	Anesthesia student	4.25 (2.01)	
	Anesthesia staff	3.44 (2.06)	
Has taken an ECG course?	*Yes*	5.53 (2.06)	t = 2.291 *p* = 0.023
	*No*	4.92 (2.33)	
Type of training	Online	5.22 (2.20)	F = 2.010 *p* = 0.136
	Face-to-face	5.20 (2.26)	
	Partial face-to-face	4.32 (2.24)	
Experience		5.13 (2.25)	*r* = −0.056 *p* = 0.319
Last training time Years since taking the course		5.94 (5.18)	*r* = 0.087 *p* = 0.119
How many times do you record an ECG during a work shift?		2.41 (1.89)	*r* = 0.094 *p* = 0.091
Is ECG interpretation one of your tasks?	*Yes*	5.47 (2.23)	t = 2.632 *p* = 0.009
	*No*	4.82 (2.24)	
Self-assessment of the ECG interpretation competency	Weak	3.84 (2.06)	F = 11.851 *p* < 0.001
	Moderate	5.33 (2.16)	
	Good	5.70 (2.26)	
	Very Good	6.38 (1.70)	
Do you have experience working in the coronary care unit (CCU)?	*Yes*	5.74 (1.76)	t = 2.624 *p* = 0.014
	*No*	4.98 (2.34)	
Type of hospital	Hospital (heart specialization)	5.81 (1.71)	F = 5.328 *p* = 0.005
	General Referral hospital	4.86 (2.47)	
	Referral hospital (nonheart specialization)	5.00 (2.06)	

### Predictors of electrocardiogram interpretation competency

The results of multiple regression analysis of predictors of electrocardiogram interpretation competence of health care professionals are shown in Table 4**.** Multiple linear regression analysis was performed using the competency of interpreting electrocardiogram as a dependent variable and demographic characteristics as independent variables. Out of 13 variables, 4 variables were significant predictors of electrocardiogram interpretation competency of healthcare professionals and students including education level (β = 0.22, R^2^ = 0.20, *p* < 0.003), self-assessment of electrocardiography interpretation competency (β = 0.19, R^2^ = 0.20, *p* = 0.001), work experience (β = − 0.19, *R*^2^ = 0.20, *p* = 0.038), and type of hospital (β = − 0.13, *R*^2^ = 0.20, *p* = 0.015).

**Table 4:** Multiple regression analysis predicting electrocardiogram interpretation competency

Variables	B	S. E	Beta	t	Sig
(Constant)	5.153	1.436		3.588	*P* < 0.0001
Age	0.504	0.280	0.166	1.797	0.073
Gender	−0.042	0.242	−0.009	−0.176	0.861
Educational Level	0.554	0.182	0.221	3.043	0.003
Job Title	−0.115	0.106	−0.078	−1.086	0.279
Experience	−0.085	0.041	−0.195	−2.082	0.038
Has taken an ECG course?	−0.335	0.268	−0.071	−1.250	0.212
Last training time Years	0.123	0.070	0.096	1.766	0.078
Type of training	−0.198	0.212	−0.048	− 0.930	0.353
Task EKG	−0.042	0.248	−0.009	−0.170	0.865
Self-assessment of EKG interpretation competency	0.586	0.170	0.197	3.443	0.001
Experience in CCU	−0.443	0.336	−0.079	−1.316	0.189
How Many EKG Record in Shift	−0.061	0.060	−0.059	−1.020	0.309
Type of Hospital	−0.458	0.186	−0.134	−2.455	0.015

*R*^2^ = 0.20, F (6.024), *P* < 0.001 A Dependent variable: Electrocardiogram interpretation competency

## Discussion

Electrocardiogram (ECG) is an essential screening and diagnostic instrument used to identify cardiovascular abnormalities as well as fatal cardiac emergencies. ECG interpretation competency is a combination of clinical knowledge, regular practice in ECG interpretation, and practical experience [1]. According to available data, this study was the first of its kind conducted to evaluate ECG interpretation competency among healthcare professionals and students in Iran.

The findings of our study show that most of the participants did not have the required ECG interpretation competency. The average score of participants was 5.3 ± 2.2 (Top score = 10), which indicates a low ECG interpretation competency. Consistent with these findings, Getachew’s study also showed low ECG interpretation competency in medical interns [23]. Also, the mean score obtained by the participants was lower than the score obtained in the study by Ho et al., which revealed a mean score of 7.7 ± 1.8 for emergency nurses [24]. The findings of another study in Iran showed that the mean score of ECG interpretation competency among emergency nurses and EMS staff was 6.65 ± 2.16 and 4.87 ± 1.81 [5]. The findings of another study in Saudi Arabia showed that 64.2% of EMS students had good ECG interpretation competency [16]. In a study by Mabuza et al., an estimated two-thirds of GPs evaluated their ECG interpretation competency as weak and only 3% evaluated it as good [13]. The reason for this low competency in our study might be that we also assessed students, as the examined medical staff scored higher compared to the students. Also, this study was conducted in November 2021, when the clinical attendance of students was limited due to the Covid-19 pandemic, preventing them from practically deepening their knowledge by experiencing critical situations. This limitation might have negatively affected our findings. Based on the results of the present study, there is a need to provide ECG interpretation training programs among participants, especially students. Ideal training should focus on demonstrating appropriate ECG measurements, which will gradually lead to a final correct diagnosis [25]. The classical approach to ECG training relies on written materials such as books and manuscripts, training courses and exercises.

A significant proportion of participants in this study (77.7%) could not detect a normal sinus rhythm. The correct answer to the corresponding question appeared to be ‘atrial bradycardia’ at first glance, but since the question stated that the patient was “athletic and thin, reporting chest pain from the time exercises end”. Therefore, it can be said that this question, having a deviation point, has caused the participants not to pay attention to the key point of the question and choose the option of bradycardia. Results of studies show that exercise can lower the heart rate [26, 27]. Therefore, the number of heartbeats given in the questionnaire was normal for athletes and was not indicative of bradycardia.

It must be emphasized that in our study, a significant number of participants were unable to detect ECG symptoms for acute myocardial infarction (ST elevation) and chronic myocardial infarction (pathological Q waves). This was consistent with the results of the study by Ahmed, Coleman, and Santos [6, 28, 29], but rather inconsistent with the results of the study by Laura [10]. Interpreting the symptoms of an acute heart attack, including ST elevation myocardial infarction (STEMI), is difficult and challenging, and most health professionals do not have the expertise to interpret these results accurately [30, 31]. The quick and correct diagnosis and interpretation of myocardial infarction can significantly reduce the door-to-balloon time for reperfusion [32, 33]. Given the urgency of this issue, finding that medical staff and students have poor competency in this respect is a cause for concern, requiring ongoing training on ECG interpretation competency to address this lack of knowledge. It is highly recommended to offer ECG interpretation training courses, especially on ST elevation MI, to healthcare staff and students. Any training must also include exposure to real-world clinical scenarios in hospital settings.

Multiple regression analysis results showed that education level was a predictor of ECG interpretation competence among healthcare professionals and students. In our study, participants with a PhD degree obtained the highest average, and participants with an associate degree obtained the lowest average in ECG interpretation competency. In Iran, the duration of a PhD course is 7 years, and the duration of an associate degree is 2 years. Therefore, the higher the educational or professional qualification of a participant, the higher will be their ECG interpretation competency. This was consistent with the findings of the previous studies [16, 17]. This may be justified by the fact that the staff and students of nursing, anesthesiology and EMS do not have to interpret ECGs on a daily basis, unlike physicians and medical students. Also, they receive more personal training and expertise in ECG interpretation [5, 32]. This is a warning sign for healthcare policymakers, who need to plan and implement useful measures to train nurses and other healthcare workers in ECG interpretation and ensure they are specialized in this task.

The results of our study showed a significant relationship between ECG interpretation competency and self-assessment among the participants. This is consistent with the studies by Kopke and Rahimpoor [5, 34]. Kopke et al. showed that medical staff and students are usually able to accurately assess their ECG interpretation competency. Those who assessed their competency as well gave more correct answers compared to those who assessed their competency as poor. Rahimpoor et al. further showed that hospital emergency nurses who assessed their ECG interpretation competency as good and very good obtained a higher average competency score [5]. It seems that individuals who are more educated and training in ECG interpretation may have more accurate interpretations due to their greater self-confidence. Today, ECG interpretation training methods must be efficient, flexible, stable, and able to take language learners from beginner to advanced translators [1]. In the modern era of technology-based medical education, online education or “e-learning” has emerged as a promising and popular educational format.

The findings of this study showed that work experience is a determinant of ECG interpretation competency. With increasing work experience, competency in ECG interpretation deteriorates. No studies were found to support this finding. In contrast, however, the results reported by Tahboub and Viljoen showed a close relationship between clinical experience and increased ECG interpretation competency [2, 19]. Nonetheless, there was no relationship between ECG interpretation competency and work experience in the findings of the study by Rahimpoor et al. [5]. Updating the knowledge and competency of health professionals in ECG interpretation to quickly identify, interpret, and intervene in rhythm abnormalities is crucial. Despite the fact that staff and students take courses on ECG interpretation in their first years of work and study to promote these skills, their inability to put these skills to practice in the long run, reduces their adroitness. This is referred to in the literature as declining skills, or “the loss or decay of trained or acquired skills (or knowledge) after periods of non-use” [4, 22]. Moreover, repeated training on an annual basis with the support of an ECG specialist can be one of the ways to achieve the level of training proven necessary for achieving a better competency in interpreting ECGs [35].

In our study, the type of hospital was another factor involved in the high points received in ECG interpretation by the participants, as the staff and students at specialty cardiac hospitals scored higher than the participants working in other hospitals. This is consistent with the findings of Chen and Rahimpoor [4, 5] and is perhaps due to the fact that staff and students at specialty cardiac hospitals only take care of cardiac patients and are more exposed to ECG interpretation during their work shifts, training hours, and internships. Therefore, they are constantly involved in ECG interpretation and enjoy a higher competency. Given that the American Heart Association states that at least 500 ECGs over a 12-month period are required to qualify for ECG interpretation, and at least 100 ECGs per year to maintain ECG interpretation skills. Therefore, recommend maximizing the exposure of medical students and healthcare staff to ECG interpretation in order to ensure their familiarization with the circumstances specified and advised by ECG training programs on the job and in clinical training.

### Strengths and limitations

An appropriate sample size is the main advantage of this study. Also, the study was conducted in all the teaching hospitals and medical emergency centers affiliated with Ardabil University of Medical Sciences with the participation of all the medical specialists who are usually involved in ECG interpretation, such as clinical physicians and students, nursing staff and students, EMS staff and students, and anesthesiology staff and students. A simple random sampling method was used to prevent bias. The study population was limited to the staff and students of the mentioned university; therefore, the findings cannot be generalized to other cities and countries. In order to actively monitor the completion of the questionnaires and increase the accuracy of the study findings, the participants were asked to answer the questions and return the questionnaire to the researcher on the spot, which means that the participants might have rushed to answer the questions. Further studies are needed to investigate the predictors of competency in ECG interpretation among healthcare professionals and students.

## Conclusion

This study is one of the first studies in the field of ECG interpretation competence among healthcare professionals and students in Iran. Our findings showed that the mean ECG interpretation score of the participants was at a low level and the knowledge of ECG interpretation, especially in the field of ST elevation MI symptoms, still needs to be significantly improved. Therefore, measures should be taken to empower them regarding ECG interpretation, and in this regard, it is necessary to hold standard training courses related to ECG interpretation and techniques to strengthen the training and learning of ECG interpretation, such as ECG display training packages. Participants’ poor competency in interpreting EKGs can also be improved by using computer-aided diagnosis and a focus on medical imaging. The results of our study revealed that many other factors also affect the interpretation of the ECG. Accordingly, presidents of medical universities are advised to consider positive self-assessment and exposure to situations where ECG interpretation is necessary when developing educational interventions to improve competency among healthcare professionals and students. For better conclusions in this regard, continuing education is essential to promote ECG interpretation competency.

## Data Availability

Data and materials can be requested from the corresponding author.
